# Adherence and Growth Outcomes in a Large Cohort of Children Treated With Recombinant GH Using a Connected Auto-injector

**DOI:** 10.1210/jendso/bvaf162

**Published:** 2025-10-22

**Authors:** Michel Polak, Natacha Bouhours-Nouet, Paula van Dommelen, Lilian Arnaud, Sophie Berger, Claire Castello-Bridoux, Quentin Le Masne, Mélissande Simonin, Raphaël Untereiner, Viviane Jeanbat, Ekaterina Koledova, Maithé Tauber, Agnès Linglart

**Affiliations:** Department of Pediatric Endocrinology, Diabetology and Gynecology, Hôpital Universitaire Necker Enfants Malades, Centre des Maladies Endocriniennes Rares de la Croissance et du Développement, Assistance Publique Hôpitaux de Paris, Paris 75015, France; INSERM U1016, Institut IMAGINE, Université Paris Cité, Paris 75015, France; Department of Paediatric Endocrinology and Diabetology, Angers University Hospital, Angers 49000, France; Department of Child Health, The Netherlands Organization for Applied Scientific Research TNO, Schipholweg 77–89, 2316 ZL Leiden, The Netherlands; Devices and Connected Solutions Engineering, Global Healthcare Operations, Ares Trading S.A. (an affiliate of Merck KGaA, Darmstadt, Germany), Eysins 1262, Switzerland; Medical Affairs Department Endocrinology, Merck Santé S.A.S., (an affiliate of Merck Healthcare KGaA, Darmstadt, Germany), Lyon 69008, France; Medical Affairs Department Endocrinology, Merck Santé S.A.S., (an affiliate of Merck Healthcare KGaA, Darmstadt, Germany), Lyon 69008, France; Connected Health & Devices Department, Akkad SAS, Lyon 69003, France; Devices and Connected Solutions Engineering, Global Healthcare Operations, Ares Trading S.A. (an affiliate of Merck KGaA, Darmstadt, Germany), Eysins 1262, Switzerland; Medical Affairs Department Endocrinology, Merck Santé S.A.S., (an affiliate of Merck Healthcare KGaA, Darmstadt, Germany), Lyon 69008, France; Quality Management Department, CEMKA, Bourg-la-Reine 92340, France; Global Medical Affairs, Cardiometabolic & Endocrinology, Merck Healthcare KGaA, Darmstadt 64293, Germany; Department of Paediatric Endocrinology, Obesity, Bone Diseases, Genetic and Gynecology, Toulouse University Hospital, Toulouse 31059, France; Paris Saclay University, Assistance Publique Hôpitaux de Paris, INSERM, Department of Endocrinology and Diabetes for Children, Bicêtre Paris-Saclay Hospital, Le Kremlin-Bicêtre 94270, France

**Keywords:** adherence, catch-up growth, connected auto-injector, growth hormone deficiency, small for gestational age

## Abstract

**Context:**

Adherence to recombinant GH treatment is crucial to achieve optimal adult height in children with growth disorders. Information on factors responsible for differences in adherence and growth in different countries is limited.

**Aim:**

To evaluate adherence and catch-up growth in children receiving GH using Easypod® auto-injectors.

**Methods:**

Retrospective cohort analysis of adherence and height, from 481 children (55% boys) in 19 centers in France, from the Growzen® Connect database, from mid-2018 to end-2023. Adherence was classified as high (≥85% of injections) or medium/low (<85%). Gain in height SD score (HSDS) was evaluated after 1 and 2 years of GH treatment.

**Results:**

High adherence was maintained throughout for 85% of patients. Among children who started young (mean age 6.3 years), 91% had high adherence over the first 2 years, significantly more than the 83% among those starting older (13.1 years) (*P* = .039). HSDS gain at 2 years for children with GH deficiency and born small for gestational age was +1.1 and +1.0, respectively, for children who started young and +0.6 and +0.6 for those starting older (*P* = .001 and.032, respectively), with no difference between disorders (*P* > .5). For children with high adherence, 2-year HSDS gain was significantly higher than for children with lower adherence (0.8 vs +0.5, *P* = .030).

**Conclusion:**

Children using Easypod exhibit high adherence, which aids clinical decision-making and patient engagement. Adherence and catch-up growth decrease with age. Indication for GH treatment had little impact, but overall catch-up growth was significantly greater with high adherence.

Short stature is caused by a wide range of health disorders. In a few conditions, including GH deficiency (GHD) and children born small for gestational age (SGA), daily subcutaneous injection of recombinant human GH is indicated to improve growth velocity and adult height. In most children, GH therapy is performed from diagnosis until completion of growth [1-3], and it is known that continuous long-term treatment is important for optimal clinical outcomes [4]. Adherence to prolonged GH therapy through daily subcutaneous injections is, therefore, a challenge. Studies in children and adolescents have shown that adherence may be suboptimal in a significant proportion of patients, affecting growth response and adult height [5-9]. Multiple causal factors are associated with low adherence, including age (in particular adolescence), socioeconomic status of patients, duration of treatment, pain during injection, and lack of information regarding consequences of missed doses [10-13]. Thus, early identification of impaired adherence and implementation of appropriate measures to overcome issues are necessary to improve patient engagement and treatment outcomes [12-14].

Digitally connected devices are becoming an integral component of healthcare, especially for chronic diseases, enabling patient/caregiver engagement and shared decision-making with healthcare professionals to improve outcomes [6, 15-17]. For pediatric growth disorders, the Growzen® Connect (formerly Easypod® Connect) ecosystem enables real-time monitoring of adherence to GH treatment [16-19]. Data collected through the connected auto-injector and apps can aid analysis of adherence and growth of patients, facilitating appropriate measures as and when required [12, 13, 20]. The global Easypod Connect Observational Study showed high adherence (≥85% of injections) for more than 3 years. In that study, adherence was significantly correlated with gain in HSDS at 1 year [14]; this was further confirmed in different countries [21, 22]. Moreover, a recent retrospective study showed that the change in catch-up growth, for up to 4 years, was significantly higher with a connected device than with a nonconnected manual device, irrespective of the indication for GH treatment [23].

The primary objective of this retrospective, observational, real-world study was to evaluate treatment adherence, according to potential explanatory variables, on a large cohort of children from multiple centers receiving GH with a connected auto-injector. Other objectives included the assessment of adherence over time, variables affecting height and catch-up growth, and the impact of adherence on catch-up growth. With an original study design, we aimed to gain knowledge of the use in France of the Easypod auto-injector and connected ecosystem to ensure that children receive the right support during their GH treatment journey.

## Materials and Methods

### Study Design and Participants

The retrospective Study and Collection of Observational data for Patients with Easypod Connect in 19 study centers in France followed children with growth disorders who received GH treatment using the Easypod auto-injector for up to 5 years. The study was conducted in compliance with the relevant international guidelines and local regulations in France, with an informed consent form signed by the parents or caregivers of the children for data collection and use of pseudonymized data. Data management processes were fully compliant with General Data Protection Regulation and cybersecurity standards and with relevant international standards, best practices, and certification.

The data for this analysis were collected from the Growzen Connect (formerly Easypod Connect) database. Information on GH injections was transmitted electronically from Easypod devices to the database, with patient demographics and clinical characteristics entered by attending clinicians. As a retrospective, observational study, all diagnoses were solely at the discretion of the attending clinicians, reflecting routine clinical practice within France. Participants included in the analysis were children with growth disorders aged 4 to 17 years treated with GH (Saizen®, Merck KGaA, Darmstadt, Germany) via the connected auto-injector Easypod between June 1, 2018, and December 31, 2023. Patients reaching 18 years of age during the study were withdrawn, with data collected before their anniversary date kept in the analysis.

Children in the study had been diagnosed with multiple disorders: GHD, born SGA, Turner syndrome, and chronic renal failure. In the main analysis, data from all children have been taken into account. However, due to the limited size of the groups of children with Turner syndrome and chronic renal failure, detailed analyses by disorders were only conducted for the GHD and SGA groups.

### Statistical Analysis

**Figure 1.**
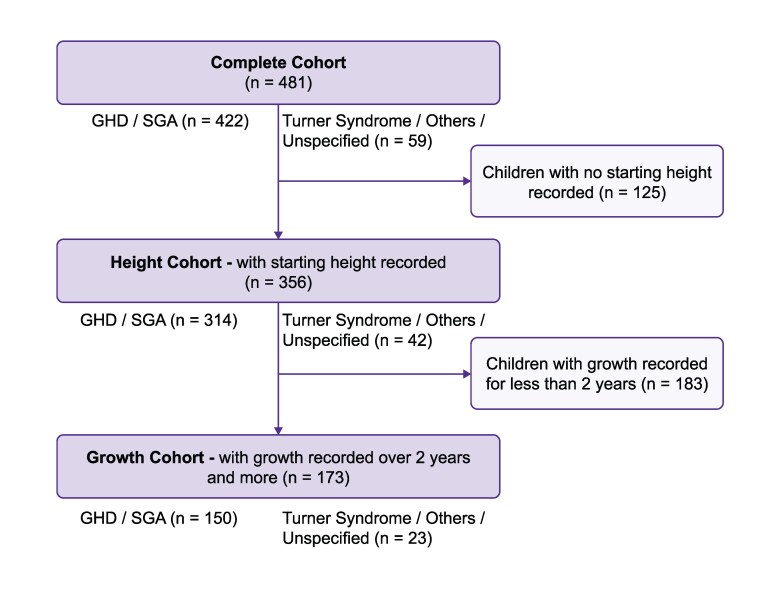
Flow chart of cohorts analyzed in the study. Within each cohort, children were also grouped by age at start as early: girls <8 years, boys <9 years; intermediate: girls ≥8 years to <11 years, boys ≥9 years to <12 years; late: girls ≥11 years, boys ≥12 years. Abbreviations: GHD, GH deficiency; SGA, small for gestational age.

The complete cohort comprised patients who used the Growzen Connect ecosystem for at least 2 weeks and had injection information recorded in the database for at least 10 days (n = 481), which was considered the minimum to allow accurate assessment of adherence (Fig. 1). Within the complete cohort, 2 subgroups were defined: the height cohort, comprising patients who had height data recorded at start of treatment (n = 356), and the growth cohort, comprising patients who had height recorded for at least 2 years (n = 173). Within each cohort, 3 groups based on age at start of treatment were defined: early (girls aged <8 years and boys <9 years), intermediate (girls ≥8–<11 years and boys ≥9–<12 years), and late (girls ≥11 years and boys ≥12 years); these groups were expected to primarily comprise patients before, around, and after the onset of puberty, respectively [24].

A formal process was established, in which information from the main database was copied to a secure controlled environment to create a pseudonymized database, with all personal data removed. Aggregated anonymized data were computed and analyzed directly. R software (V4.0.3 and further) and Python (Anaconda Distribution Platform, latest version) were used for data management and statistical analysis. A predefined concept generation analysis was conducted to address the clinical objectives.

Adherence was computed as mean weekly adherence and categorized as high (≥85% of injections), medium (>56–84%), or low (≤56%), consistent with other studies where high adherence corresponds to less than 1 missed injection per week on average [5, 9, 19]. Height SD score (HSDS) was calculated using French reference growth curves [25], with catch-up growth defined as the HSDS change from GH treatment start to the relevant time point. GH dose was calculated and set in the Easypod device by the attending clinician, according to approved guidance for the indication. Since GH can be prescribed either as 7 injections per week or 6 injections per week (with a 1-day pause), we normalized the prescribed dose (the dose set in the auto-injector before injection) to facilitate comparison, and we computed the normalized prescribed daily dose by body weight starting from the total weekly dose. The mean normalized prescribed daily dose by body weight was calculated as the total prescribed dose by body weight over a week divided by 7.

Descriptive analyses were performed, and, where relevant, variables were stratified by year/month, sex, age, and standardized height at study start. Differences in baseline parameters (eg, HSDS) between the age groups were calculated and evaluated by *t*-test statistics, with statistical significance established for *P-*values below .05, while differences in proportions of children with a high adherence level were evaluated by 2-sample *z*-tests (with adjustment for age where relevant). The independent effect of age groups, HSDS at start of treatment, disorders, and level of adherence on catch-up growth was evaluated using linear regression analysis using the gain in HSDS over the first 2 years as outcomes.

## Results

### Patient Population

The distributions of patients according to demographics and disorders are shown in Table 1. The complete cohort of 481 patient (55% boys, 45% girls) had a mean age of 9.9 (SD = 2.9) years at treatment start, with a mean age at treatment start of 9.3 (2.5) years for girls (min: 4.1 years, max: 15.1 years) and 10.4 (3.2) years for boys (min: 4.0 years, max: 16.6 years). The recorded duration of the use of Growzen Connect was more than 3 months for 97% of the patients, and the median duration was greater than 2 years. The reasons for GH treatment were predominantly GHD for 265 (55%) patients and born SGA for 157 (33%) patients. The demographics and distribution of disorders were similar for the height cohort (n = 356), and the mean HSDS was –2.4 (SD = 0.8). The growth cohort (n = 173) included slightly fewer patients with GHD (49%) and more born SGA (38%), with a similar deficit in mean HSDS at treatment start (−2.5, SD = 0.7).

**Table 1.** Baseline characteristics of the study analysis sets

Parameter	Category	Complete cohort (n = 481)	Height cohort (n = 356)	Growth cohort (n = 173)
Sex, n (%)	Boys	265 (55)	200 (56)	93 (54)
	Girls	216 (45)	156 (44)	80 (46)
Age at start (years), mean (SD)		9.9 (2.9)	9.9 (2.9)	9.7 (2.7)
	Boys	10.4 (3.2)	10.5 (3.0)	10.3 (3.0)
	Girls	9.3 (2.5)	9.3 (2.5)	9.0 (2.2)
HSDS at start, mean (SD)		NA	−2.4 (0.8)	−2.5 (0.7)
	Boys	NA	−2.3 (0.8)	−2.5 (0.8)
	Girls	NA	−2.4 (0.8)	−2.4 (0.7)
Indication n (%)	GHD	265 (55)	207 (58)	85 (49)
	SGA	157 (33)	107 (30)	65 (38)
	Turner syndrome	13 (3)	10 (3)	7 (4)
	Other/unspecified	46 (10)	32 (9)	16 (9)
Boys/girls by indication, n/n	GHD,	157/108	128/79	50/35
	SGA	82/75	54/53	34/31
	Turner syndrome	0/13	0/10	0/7
	Other/unspecified	26/20	18/14	9/7

Complete cohort comprised patients with ≥10 days of injection data available; height cohort comprised patients with height measurements at study start; growth cohort comprised patients with height data recorded for at least 2 years.

Abbreviations: GHD, GH deficiency; HSDS, height SD score; NA, not available; SGA, small for gestational age.

In the complete cohort overall, 47% were using a 6 injections/week regimen, with 53% using 7 injections/week, whereas worldwide data indicate 15% and 85% for 6 and 7 injections/week, respectively (from an unpublished analysis performed in parallel on worldwide data from Growzen Connect using similar methods). However, the distribution between the 2 regimens was heterogeneous among the French study centers, ranging from 4% to 97% for the proportion of children using a 6 injections/week regimen. This high variation was not explained by differences in ages or indications of the population but most likely depended on the standard practice within different clinical centers. For children with GHD, the mean normalized daily prescribed GH dose was 0.039 and 0.035 mg/kg per day for the 6 and 7 injections/week regimens, respectively, and as expected, was slightly higher for children born SGA with a mean normalized dose of 0.045 and 0.041 mg/kg per day, respectively, with no difference according to sex.

### Adherence

**Figure 2.**
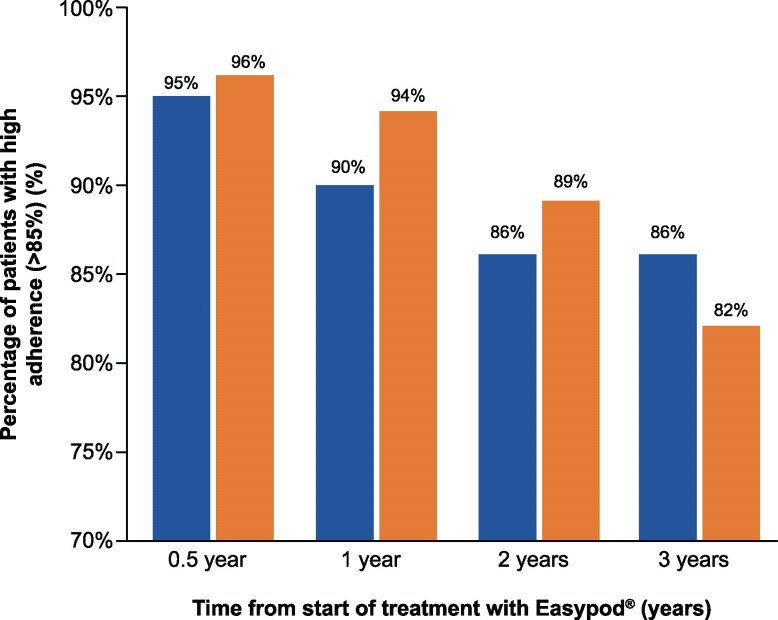
Proportion of children with high adherence as a function of time from start of treatment with Easypod for children with GHD and children born SGA. For GHD, n = 254 at 0.5 year, 231 at 1 year, 132 at 2 years, 62 at 3 years. For SGA, n = 153 at 0.5 year, 144 at 1 year, 108 at 2 years, 61 at 3 years. Abbreviations: GHD, GH deficiency; SGA, small for gestational age.

Throughout the entire analysis period, the adherence level was high for 85% of patients, medium for 13%, and low for 2%. Overall median (Q1/Q3) adherence was 99% (97%/100%), indicating that at least half of the patient population had adherence above 99%, corresponding to less than 4 injections missed every year. The percentage of patients with high adherence over time is shown in Fig. 2 for children with GHD and born SGA. In both groups, adherence tended to drop over time, but more than 80% of patients still had a high adherence level at 3 years.

Among the group of children with an early starting age (mean 6.3 years), 91% had high adherence over the first 2 years after treatment start. This proportion dropped to 88% among children with an intermediate starting age (mean 10.1 years) and to 83% for children with a late starting age (mean 13.1 years) (*P* = .039 when comparing late to early groups), indicating that age at the time of treatment had a significant impact on the level of adherence.

Assessment of the proportion of children with a high adherence level according to injection regimen (6 or 7 injections/week) over the study period indicated no significant difference after correction for age at treatment start (*P* > .10).

### HSDS at Start

HSDS at the start of treatment, evaluated according to the height cohort starting-age groups, was −2.7 (SD = 0.7) for the early group, −2.3 (0.7) for the intermediate group, and −2.1 (0.8) for the late group (*P* < .05 when comparing each group with another), indicating a greater height deficit for patients who started GH at a younger age (Table 2). Height deficit was significantly greater for the children born SGA than those diagnosed with GHD overall (HSDS −2.7 vs −2.2, *P* < .001) and for each of the starting-age groups.

**Table 2.** Characteristics of patients within the height cohort, stratified by starting age groups and growth disorder

	All	Early	Intermediate	Late
Total				
n	356	101	144	111
Boys, n (%)	200 (56.2)	56 (55.4)	71 (49.3)	73 (65.8)
Age at start, years	9.9 (2.9)	6.3 (1.3)	10.1 (0.9)	13.1 (1.2)
HSDS at start	−2.4 (0.8)	−2.7 (0.7)	−2.3 (0.7)*	−2.1 (0.8)**
GHD only				
n	207	48	85	74
Boys, n (%)	128 (61.8)	33 (68.8)	44 (51.8)	51 (68.9)
Age at start, years	10.3 (2.7)	6.5 (1.4)	10.1 (1.0)	13.1 (1.1)
HSDS at start	−2.2 (0.7)	−2.5 (0.7)	−2.2 (0.6)*	−2.1 (0.9)*
SGA only				
n	107	46	40	21
Boys, n (%)	54 (50.5)	22 (47.8)	19 (47.5)	13 (61.9)
Age at start, years	8.9 (2.9)	6.1 (1.3)	10.2 (0.9)	12.8 (1.0)
HSDS at start	−2.7 (0.7)***	−3.0 (0.6)	−2.6 (0.7)*	−2.3 (0.6)**

Values are presented as mean (SD). Starting-age groups are early: girls <8 years, boys <9 years; intermediate: girls ≥8 years to <11 years, boys ≥9 years to <12 years; late: girls ≥11 years, boys ≥12 years.

Abbreviations: GHD, GH deficiency; HSDS, height core; SGA, born small for gestational age.

^*^ *P* < .05 compared with early; **P < .05 compared with early and intermediate, ***P < .001 for SGA compared with GHD overall.

**Figure 3.**
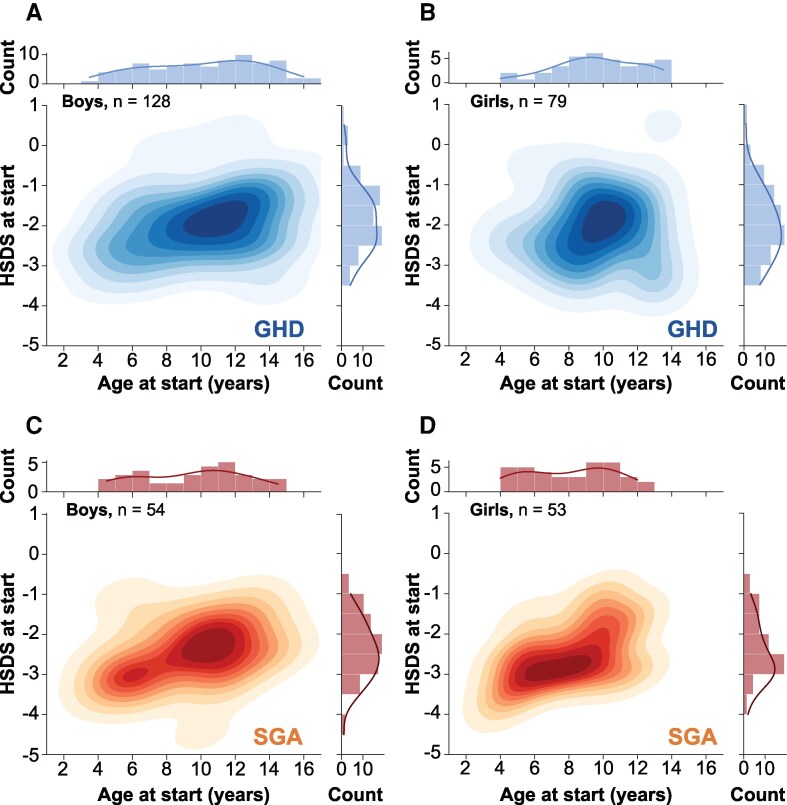
Distribution of boys (left) and girls (right) in the height cohort as a function of age at start (years) and HSDS at start, by indication of GHD (top; blue) and SGA (bottom; orange). Abbreviations: GHD, GH deficiency; HSDS, height SD score; SGA, small for gestational age.

The distribution of HSDS by age at the start of treatment for children with GHD and born SGA for boys and girls is shown in Fig. 3, using color intensity to indicate the size of the population. In all cases, patients starting at a younger age tended to have a lower HSDS. Additionally, Fig. 3 indicates a lower HSDS and a younger age at treatment start for the SGA cohort than the GHD cohort, especially for girls.

### Growth Outcomes

**Figure 4.**
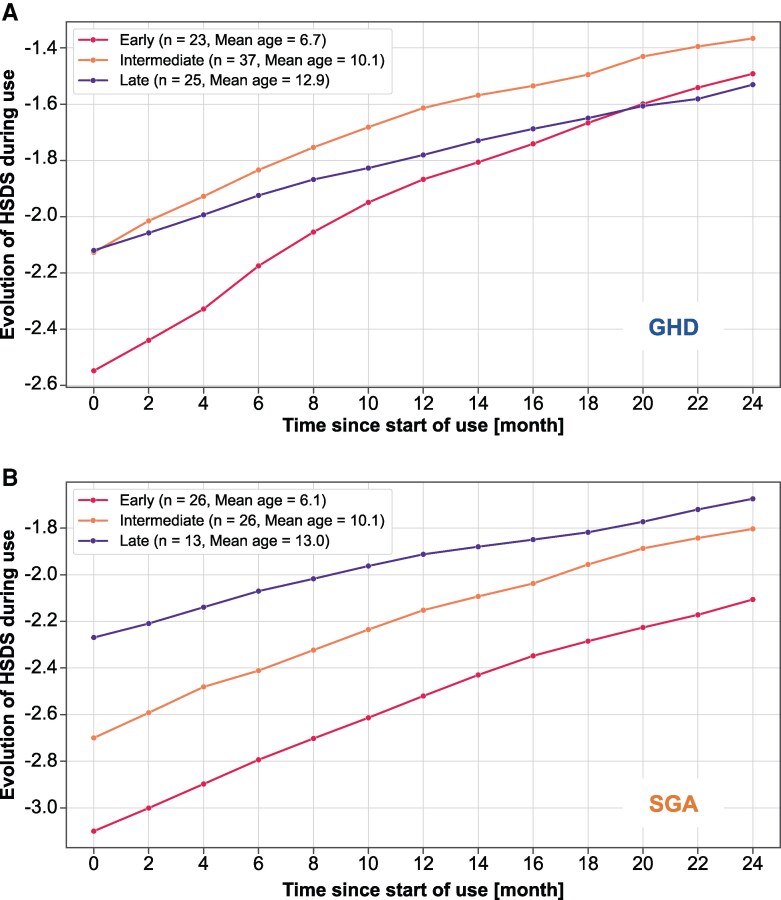
Change in HSDS for children in the growth cohort with GHD (A) and for children born SGA (B) during GH treatment using an Easypod auto-injector. Abbreviations: GHD, GH deficiency; HSDS, height SD score; SGA, small for gestational age.

Evolution of HSDS over the first 2 years of treatment is shown in Fig. 4 for each of the starting-age groups in the growth cohort for children with GHD and born SGA. HSDS gain at 1 and 2 years is shown in Table 3 for each group. Linear regression analysis indicates that better outcomes in terms of gain in HSDS over the first 2 years were associated with the following independent variables: a lower HSDS at treatment start (*P* < .001), a younger age at treatment start (*P* = .001), and a higher level of adherence (≥85%) (*P* = .030). Children in the early starting-age group had a significantly better catch-up growth at 2 years (+1.1 for GHD, + 1.0 for SGA) than children from the late group (+0.6 for GHD, + 0.6 for SGA) (*P* = .001 for GHD and *P* = .032 for SGA). The difference in HSDS gain at 2 years between the early and late starting-age groups was 0.5 and 0.4 for children with GHD and born SGA, respectively, which corresponds to a reduced height of ∼2.5 to 2.9 cm for girls and ∼3.4 to 3.9 cm for boys at an older age. The HSDS gain after 2 years was not significantly different between children with GHD and children born SGA (*P* = .612).

**Table 3.** Growth response for the growth cohort, stratified by starting-age groups and disorder

	All	Early	Intermediate	Late
Total				
n	173	54	73	46
Age at start, years	9.7 (2.7)	6.4 (1.3)	10.1 (0.9)	12.9 (1.0)
HSDS at start	−2.5 (0.7)	−2.9 (0.6)	−2.3 (0.7)	−2.2 (0.7)
Gain in HSDS at 1 year	0.5 (0.3)	0.6 (0.3)	0.5 (0.3)	0.3 (0.3)
Gain in HSDS at 2 years	0.8 (0.5)	1.0 (0.5)	0.8 (0.4)	0.6 (0.4)
*P*-value for gain in HSDS at 2 years			.058 vs early	.001 vs early .060 vs intermediate
GHD only				
n	85	23	37	25
Age at start, years	10.0 (2.6)	6.7 (1.3)	10.1 (1.0)	12.9 (0.9)
HSDS at start	−2.2 (0.7)	−2.6 (0.6)	−2.1 (0.7)	−2.1 (0.7)
Gain in HSDS at 1 year	0.5 (0.3)	0.7 (0.4)	0.5 (0.3)	0.3 (0.2)
Gain in HSDS at 2 years	0.8 (0.5)	1.1 (0.5)	0.8 (0.4)	0.6 (0.4)
*P*-value for gain in HSDS at 2 years			.037 vs early	.001 vs early .11 vs intermediate
SGA only				
n	65	26	26	13
Age at start, years	9.1 (2.9)	6.1 (1.4)	10.1 (0.8)	13.0 (1.0)
HSDS at start	−2.8 (0.7)	−3.1 (0.5)	−2.7 (0.7)	−2.3 (0.5)
Gain in HSDS at 1 year	0.5 (0.3)	0.6 (0.3)	0. 5 (0.2)	0.4 (0.3)
Gain in HSDS at 2 years	0.9 (0.4)	1.0 (0.4)	0.9 (0.3)	0.6 (0.5)
*P*-value for gain in HSDS at 2 years	> .5 vs GHD		> .5 vs early	.032 vs early .057 vs intermediate

Abbreviations: GHD, GH deficiency; HSDS, height SD score; SGA, small for gestational age.

Values are presented as mean (SD), with *P*-values calculated by regression analysis. Starting-age groups are early: girls <8 years, boys <9 years; intermediate: girls ≥8 years to <11 years, boys ≥9 years to <12 years; late: girls ≥11 years, boys ≥12 years.

HSDS gain after 2 years was +0.9 for children using 6 injections/week (n = 88) and +0.7 for children using 7 injections/week (n = 85). The slightly higher gain for the 6 injections/week regimen was consistent with a lower mean age at treatment start (8.9 years vs 10.5 years).

Over 2 years of GH treatment, 154 patients from the growth cohort showed a high (≥85%) level of adherence, whereas 19 patients showed medium/low (<85%) adherence. At the start of treatment, the mean age was 9.5 years and 10.9 years, respectively, and the mean HSDS was –2.5 and –2.2, respectively. The HSDS gain was better for the high adherence group after 1 year and 2 years (+0.5 and +0.8, respectively) compared with the medium/low adherence group (+0.3 and +0.5, respectively). Regression analysis with adjustment for age and HSDS at treatment start indicated that level of adherence (high vs medium/low) had a significant impact on 2-year HSDS gain (*P* = .030).

## Discussion

This study showed that using a connected auto-injector for GH injections in children with growth disorders maintained high adherence over time, which was associated with improved catch-up growth. Adherence and height gain data were analyzed from a large cohort of children treated with daily GH in 19 clinical centers distributed throughout France. Data were collected in a database through the digitally connected Easypod auto-injector and Growzen Connect ecosystem [18, 19]. As a retrospective, observational study, the information can be considered representative of the current real-world population of children eligible for GH therapy in France in terms of distribution of disorders, starting age, and sex distribution [17, 26]. The primary endpoint was treatment adherence rate evaluated in the complete cohort, which was maintained at a high level for at least 3 years. The overall high level of adherence (≥85% for 85% of children) was at least as good as in similar studies conducted with children using the Easypod device in other countries and confirmed good engagement of patients with their treatment [14, 21, 22].

Adherence to GH treatment is frequently suboptimal, decreasing over time due to factors such as patient fatigue, gradual self-management in the teenager years, lack of education about the consequences of missing doses, and poor patient-clinician relationships [27-30]. Consistent with this, age at the time of treatment had a profound effect on adherence in the present analysis, with a decrease observed at adolescence. High adherence has been shown to be maintained with connected auto-injectors and is influenced by patients’ perceptions of their features and utility [23, 30-33]. However, the appropriate approach to help children achieve high adherence rates will depend on sociocultural and economic circumstances and may rely more on patients' preferences and interpersonal relationships than technical solutions. Increased therapeutic education, provided directly by centers and/or through apps incorporated in the Growzen Connect ecosystem also aids good adherence [11, 16, 18, 19]. The latest generation of Easypod, launched in 2023, with improved features including enhanced ease of use and ergonomics, reduced needs for battery charge, and automatic transmission of recorded data potentially strengthens support in the event of patient fatigue [33, 34].

Our results demonstrated that both regimens, using 6 or 7 injections/week, led to the same levels of adherence (yet with a slightly higher daily dose in the 6-days regimen, possibly due to rounding errors when calculating the dose). This is important because both regimens seem evenly used in France for the treatment of short stature in children with GHD or children born SGA. The wide variations between clinical centers likely reflect the philosophy of care and routine practices of individual physicians. Patients treated for the indication of SGA received a higher mean daily dose per body weight than those treated for GHD, as expected in France due to local clinical practices following outcomes of early clinical studies on dosing for GH [35]. Differences of this nature have been observed in other studies [26], and the doses administered remained consistent with French guidelines.

As expected, starting height deficit was greater for patients starting at an early age, since prompt diagnosis and orientation toward a pediatric endocrinologist is more likely in the case of strong deficits. However, the relationship between height deficit and starting age depends on sex and diagnosis. For boys, the most likely age at treatment start and height deficit appeared similar for children with a diagnosis of GHD and those born SGA, whereas girls born SGA started at a younger age and had a greater height deficit than girls diagnosed with GHD. For children with GHD, boys had a much broader range of age at treatment start than girls. Both the youngest and oldest patients were boys, suggesting that boys are likely diagnosed earlier for a similar height deficit than girls. Also, some boys may have the opportunity to start treatment later, because puberty may impose less of a limitation on growth.

Starting height deficit was also greater for patients born SGA than with GHD, in agreement with French recommendations and reimbursement policies for children born SGA. The mean gain in HSDS in the first year was +0.6 for children starting GH at mean 6.4 years. According to pediatric studies of GH treatment, this initial gain can be considered as a good response, which has been defined as HSDS gain >+0.5 in prepubertal children [36-38]. The gain was +0.3 for those starting at an older mean age of 12.9 years, although the late starting-age group was most likely in puberty, which could have limited the growth response to exogenous GH; however, the observed mean gain may also be considered a good response for children starting when postpubertal [37-39]. The 1-year HSDS gain was similar for the children with GHD and born SGA overall and for each starting-age group, whereas others have reported a greater catch-up growth in children with GHD than those born SGA, most likely due to differences in starting height deficit [36, 40]. However, the diagnosis was entirely at the discretion of the attending physician, with no predefined cut-off for GH stimulation tests; thus, it was possible that some patients with a diagnosis of GHD may have had non-GHD short stature and could have influenced the difference in growth response.

Mean HSDS gain after 2 years for the early and late starting-age groups was +1.1 and +0.6 for children with GHD and +1.0 and +0.6 for children born SGA. A 2-year response in HSDS gain >+0.65 for prepubertal children was reported to provide a good prediction of adult height outcome [41]. The growth difference between the early and late starting ages equated to a height difference of 2.5 to 3.9 cm, depending on sex and age, and confirms the strong impact of an early start on overall catch-up growth. The 2-year catch-up growth for the 2 injection regimens (6 or 7 injections/week) showed little difference, indicating no effect on growth benefit.

Mean HSDS gain has also been shown to be reduced with lower adherence [1, 5, 7]. In the present study, children with high adherence had a significantly greater 2-year height gain than children with lower adherence (+0.8 vs + 0.5). This was consistent with other studies using the connected ecosystem for GH administration, where good adherence was associated with a significantly better response to treatment [14, 21-23], which confirms the importance of treatment adherence in the early optimization of catch-up growth.

The study has several limitations, particularly relating to the observational nature, which limited the data set, although this reflected standard clinical practice. While Easypod automatically records all injection times, providing accurate objective evaluation of adherence, assessment in studies with other GH formulations have used self-reported methods, such as vial return or questionnaires. These are known to be inaccurate, meaning that effect size of adherence on growth would be incorrect and, thus, not comparable with the current study [1, 5, 8, 9]; there are no comparable published studies with other GH administration devices that would allow direct comparisons, although new devices capable of recording injection dates may become available in the future, potentially facilitating comparative studies. The database did not include all potential confounders, such as whether patients were naïve to GH treatment; children may have received GH before switching to treatment with the connected device but could still be termed start of use. There were also no data available on the children's education or understanding of their condition or therapy, and no socioeconomic information or medical history was available, which could have influenced adherence with treatment. It was also not known whether children who dropped out did so because of adherence or growth problems, resulting in only children willing to transmit and share data being included; however, studies with the new generation of Easypod auto-injector that automatically transmits data have shown similar adherence rates to children using auto-injectors requiring active transmission [33, 34]. The retrospective nature also meant that the data were incomplete, and the growth analysis could not take into account the entire study population; consequently, height gain across 2 years was available for only part of the complete analysis set, although sufficient data were available to provide statistically significant results. In addition, it was not possible to collect data such as cause and severity of GHD, parental height, bone age, or pubertal stage, which are factors that affect growth response and would have enabled a better clinical and medical interpretation of the results. Such factors may be used to assess growth potential and guide the decision to initiate treatment but would require a dedicated study to assess any impact on adherence and response and were considered unlikely to affect the conclusions. However, the patient population was selected as broadly as possible, including multiple centers and a wide range of patient demographics and clinical characteristics, with the aim to minimize the risk of potential bias. Additionally, the stratification by age at the start of treatment served as a relevant proxy for pubertal stage.

Despite these limitations, this study has significant strengths in terms of number of patients, diversity of centers, and duration of recorded use, which makes it relevant and brings further evidence to the existing literature. The study included a representative number of children from 19 diverse clinical centers across France, and the accuracy of the adherence data for these children could be determined through the Easypod auto-injector and Growzen Connect ecosystem.

## Conclusion

This study represents 1 of the largest analyses of real-life data collected in France on children receiving GH treatment for growth disorders. It provides better characterization of the population treated, in accordance with approved indications, and highlights specific clinical practices in France. The extremely high adherence rate shows the increasing value of a connected auto-injector for treating and monitoring patients, aiding clinical decision-making, patient engagement, and a good patient-clinician relationship. Adherence rate and catch-up growth were higher in younger patients, emphasizing the need for early initiation of treatment and, before that, for early diagnosis. Catch-up growth was greater in patients with high adherence, underscoring the importance of adherence to treatment for attaining the desired linear growth until adult height.

## Data Availability

Any requests for data by qualified scientific and medical researchers for legitimate research purposes will be subject to the Data Sharing Policy of the healthcare business of Merck KGaA, Darmstadt, Germany. All requests should be submitted in writing to the data sharing portal for the healthcare business of Merck KGaA, Darmstadt, Germany https://www.emdgroup.com/en/research/our-approach-to-research-and-development/healthcare/clinical-trials/commitment-responsible-data-sharing.html. When the healthcare business of Merck KGaA has a co-research, co-development, or co-marketing or co-promotion agreement, or when the product has been outlicensed, the responsibility for disclosure might be dependent on the agreement between parties. Under these circumstances, the healthcare business of Merck KGaA will endeavor to gain agreement to share data in response to requests.
